# Addressing student challenges in poland: policy recommendations for enhancing academic success

**DOI:** 10.1192/j.eurpsy.2026.10697

**Published:** 2026-07-31

**Authors:** A. Baran, A. Zalewska-Janowska, A. Gmitrowicz, P. Waszak, D. Biechowska, T. Milewicz, P. Stecz, J. Hawgood

**Affiliations:** 1 Karlstad University, Sweden, Sweden; 2 Medical University of Lodz, Lodz; 3 Medical University of Gdańsk, Gdansk; 4 Dept of Psychology, SWPS University, Sopot; 5 Jagiellonian University, Krakow; 6 Institute of Psychology, Lodz, Poland; 7 Griffith University, Brisbane, Australia

## Abstract

**Introduction:**

Suicide is a leading cause of death in young people, and university students show substantial prevalence of suicidal thoughts and behaviors (Mortier et al. *J Am Acad Child Adolesc Psychiatry* 2018;57:263–273). In Europe, high psychological distress and unmet support among students—conditions linked to suicide risk—have been repeatedly documented (Ochnik et al. *Sci Rep* 2021;11:18644). This study quantifies equity-related challenges in Polish students and tests their association with academic struggles to inform prevention-focused policy.

**Objectives:**

This study aims to: 1. Identify the key challenges affecting university students in Poland. 2.Analyze the statistical relationship between these challenges and academic struggles. 3. Propose data-driven policy recommendations to enhance student support services.

**Methods:**

Using a mixed-method approach, data were collected from 303 university students in Poland (January 2025) via selected questions of HESP-CCB survey created as part of the IASP Special Interest Group Education and Training in Suicide Prevention multicountry initiative, with quantitative analysis performed through descriptive statistics, chi-square tests, and logistic regression modeling. Qualitative responses were examined using thematic analysis to highlight key concerns.

**Results:**

Quantitative data highlighted financial hardship (17.5%), living in rural or remote areas (16.2%), disabilities (5.6%), LGBTQIA2S+ identity (4.3%), and an immigration background (1.7%) as relevant challenges. The most commonly selected response was “None of the above” (51.5%), indicating that over half of the students did not identify with the predefined categories. The response “Other” (10.2%) suggested the existence of additional, unlisted barriers. Thematic analysis of qualitative data emphasized mental health challenges, academic pressure, and the need for stronger institutional support.

**Conclusions:**

Based on the above findings, ten targeted policy recommendations are proposed including enhancing university support systems, improving mental health literacy and services, increasing financial aid opportunities, and introducing flexible academic policies. Addressing these challenges is crucial for improving student retention and academic success.

**Disclosure of Interest:**

None Declared

